# Laboratory screening and field validation of *Myxococcus fulvus* and *Cystobacter fuscus* for the biocontrol of wheat Fusarium Crown Rot

**DOI:** 10.3389/fmicb.2026.1804789

**Published:** 2026-04-10

**Authors:** Xiaoli Wang, Zilin Wang, Shuqi Mao, Benzhong Fu, Deying Ma, Jian Han

**Affiliations:** 1 Department of Plant Pathology, College of Agronomy, Xinjiang Agricultural University, Urumqi, China; 2 Key Laboratory of Prevention and Control of Invasive Alien Species in Agriculture and Forestry of the North-western Desert Oasis (Co-construction by Ministry and Province) Ministry of Agriculture and Rural Affairs, Urumqi, China; 3 Key Laboratory of the Pest Monitoring and Safety Control of Crops and Forests of the Xinjiang Uygur Autonomous Region, Urumqi, China

**Keywords:** biological control, Fusarium Crown Rot, *Fusarium* spp., growth promotion, myxobacteria

## Abstract

Fusarium Crown Rot (FCR), primarily caused by *Fusarium* species, is a significant soil-borne disease that severely threatens global wheat production and food security. To identify myxobacterial resources with biocontrol potential against FCR, we screened 81 myxobacterial strains using dual-culture assays and evaluated their efficacy through greenhouse and field experiments. Three strains—HM-E, KT23, and KE15—were identified for their potent broad-spectrum activity against the dominant pathogens *F. pseudograminearum*, *F. graminearum*, and *F. culmorum*, achieving mycelial inhibition rates ranging from 82.01 to 94.84%. Their cell-free filtrates significantly inhibited hyphal growth while inducing spore lysis and suppressing germination. These strains employed a range of inhibitory mechanisms complementary to their conventional predatory activity. Greenhouse trials demonstrated that both fermentation broths and solid agents provided control efficacies of 52.94–88.24% against single and complex infections, consistently outperforming the chemical fungicide Tebuconazole (40.75–59.15%). Furthermore, myxobacterial treatments significantly promoted wheat growth, as evidenced by increased plant height, primary root length, and fresh weight. In field trials, the biocontrol efficacy reached 61.60–74.67% at the flowering stage and 57.95–71.53% at the grain-filling stage. Compared to the pathogen-only control (5,860.00 kg/ha), myxobacterial treatments increased grain yield by 13.71–27.82%. Based on morphological and multigene phylogenetic analyses, strains KT23 and KE15 were identified as *Myxococcus fulvus*, while HM-E was identified as *Cystobacter fuscus*. This study identifies *C. fuscus* HM-E and *M. fulvus* KT23 and KE15 as robust biocontrol resources with dual functions in disease suppression and growth promotion, providing a novel technical framework and microbial candidates for the sustainable management of wheat Fusarium Crown Rot.

## 1 Introduction

Fusarium Crown Rot (FCR) is a devastating soil-borne disease caused by a complex of pathogenic fungi within the *Fusarium* genus, and it has become one of the most significant constraints to wheat production in arid and semi-arid regions globally (Kazan and Gardiner, 2018). First identified in Australia in the 1950s, FCR has since spread to numerous countries and regions, including the United States (Laraba et al., 2025), Kyrgyzstan, North Africa (Tanane et al., 2026), New Zealand (Aoki et al., 2015), and China (Zhou et al., 2019). In recent years, the prevalence of FCR in China has intensified due to changes in cultivation practices—such as the widespread adoption of crop rotation with other gramineous plants and straw returning—alongside climatic shifts like global warming. Currently reported across major wheat-producing provinces including Henan, Hebei, and the Northwest, FCR has emerged as a primary threat to national wheat security (Zhang et al., 2015).

The disease manifests as necrosis and decay of the seeds, seedlings, roots, and particularly the crown and stem base. This tissue damage obstructs the transport of water and nutrients, leading to typical yield losses of 10%–20%, with severe infections resulting in losses exceeding 50% (Pu et al., 2025). Beyond yield reduction, the infecting pathogens produce various mycotoxins, most notably deoxynivalenol (DON), which pose a grave threat to human and livestock health while significantly degrading wheat quality (Palacios et al., 2017; Li et al., 2024).

The etiology of FCR involves a diverse range of *Fusarium* species, including *F. pseudograminearum*, *F. graminearum*, *F. culmorum*, *F. equiseti*, *F. fujikuroi*, *F. proliferatum*, *F. oxysporum*, and *F. acuminatum* (Ozer et al., 2023; Ma et al., 2024; Zhang et al., 2025). Among these, *F. pseudograminearum*, *F. graminearum*, and *F. culmorum* are recognized as the dominant pathogens across Chinese wheat-growing regions (Zhang et al., 2015; He et al., 2025).

Current management of FCR relies primarily on a combination of cultural practices and chemical fungicides, alongside ongoing research into resistant variety screening and biological control. However, there is a notable scarcity of immune or highly resistant germplasm. Furthermore, the excessive application of chemical fungicides has led to increased pathogen resistance and raised significant environmental and food safety concerns (Meng et al., 2022; Zhou et al., 2024). Consequently, biological control has gained widespread attention as a safe, eco-friendly, and sustainable alternative.

Nevertheless, the majority of reported biocontrol strains and formulations have focused on single pathogens, particularly *F. pseudograminearum* (Feng et al., 2023). This single-target approach fails to account for the multi-pathogen complex nature of FCR, leading to biocontrol agents with a narrow spectrum of activity. Such agents often exhibit limited potential and unstable efficacy when faced with the complex multi-pathogen environments of actual fields. Therefore, there is an urgent need to develop novel biocontrol agents capable of simultaneously targeting multiple dominant *Fusarium* species to achieve broad-spectrum and high-efficiency control (Meng et al., 2024).

Myxobacteria are a unique group of social bacteria that actively hunt other microorganisms through “wolf-pack-style” predation and gliding motility. They produce a vast array of secondary metabolites and hydrolytic enzymes (Antunez Dominguez et al., 2025). Beyond direct antagonism, myxobacterial predation shapes the soil micro-ecology, playing a pivotal role in maintaining microbial balance and plant health (Dai et al., 2025). While myxobacteria have demonstrated potential in controlling diseases such as fire blight (Han et al., 2024), pepper anthracnose (Yun, 2014), tomato bacterial wilt (Dong et al., 2021), and cucumber Fusarium wilt (Ye et al., 2020b), their application in major field grain crops like wheat remains largely unexplored.

To explore the biocontrol potential of myxobacteria against FCR, this study utilized the laboratory’s myxobacterial germplasm resources to screen for strains with high antagonistic activity against the dominant pathogens *F. pseudograminearum*, *F. graminearum*, and *F. culmorum*. We further evaluated their biocontrol efficacy through greenhouse and field plot experiments to provide a basis for the development of broad-spectrum microbial agents for wheat health.

## 2 Materials and methods

### 2.1 Strains, media, and wheat varieties

A total of 81 myxobacterial strains were used in this study. These strains were previously isolated and purified from various environments in Xinjiang, China (including farmlands, forests, and the Gobi Desert) using rabbit manure and prey-induction methods (Dou et al., 2023). Notably, strain HM-E, identified as *Cystobacter fuscus*, was isolated from a cotton field infested with Verticillium wilt in Moyu County, Hotan Prefecture, Xinjiang (Han et al., 2025).

The fungal pathogens *Fusarium pseudograminearum* (*Fpg*) and *F. graminearum* (*Fg*) were kindly provided by Prof. Yancun Zhao (Institute of Plant Protection, Jiangsu Academy of Agricultural Sciences), and *F. culmorum* (*Fc*) was provided by Assoc. Prof. Lili Wang (College of Agronomy, Xinjiang Agricultural University).

Myxobacterial activation and antagonism assays were performed on VY/2 medium, while liquid cultivation was conducted in LBS medium. Fungal pathogens were cultured on Potato Dextrose Agar (PDA), and spore production was induced in Carboxymethyl Cellulose (CMC) medium. The compositions of VY/2, LBS, and PDA media were based on Nair et al. (2019), and CMC medium followed Tang et al. (2025).

The wheat varieties used were “Xindong 18” (a mid-maturity winter wheat) provided by Dr. Yukun Cheng (Cao et al., 2021) and “Hechun 137” (a mid-early maturity spring wheat) provided by Dr. Shiren Sun (Zhao et al., 2021).

### 2.2 Antagonistic activity assays

#### 2.2.1 Preparation of myxobacterial and pathogenic suspensions

After activation on VY/2 plates for 5 days, myxobacterial biofilms were inoculated into LBS broth and cultured at 30 °C and 180 rpm for 2 days. Subsequently, a 3% (v/v) inoculum was transferred to 200 mL of LBS broth and cultured for an additional 3 d under the same conditions. Cells were harvested by centrifugation (12,000 rpm, 1 min), washed three times with sterile water, and resuspended in sterile water to achieve a final OD_600_ of 2.0.

For fungal spore preparation, *Fpg*, *Fg*, and *Fc* were activated on PDA. Five mycelial plugs (5 mm diameter) were inoculated into 100 mL of CMC medium and cultured at 26 °C and 180 rpm for 7 days. The suspension was filtered through four layers of sterile gauze and adjusted with sterile water to a concentration of 1.0 × 10^6^ spores/mL.

#### 2.2.2 Mycelial growth inhibition assay

A 5-mm fungal plug was placed in the center of a VY/2 plate. At four points 2 cm away from the center, 20 μL of the myxobacterial suspension was streaked in four short lines. Plates were incubated at 28 °C, with plates inoculated only with fungal plugs as controls. Each treatment was performed in triplicate, and the experiment was repeated twice independently. After 5 d, colony areas were measured and the inhibition rate (%) was calculated as follows (Pellan et al., 2020):

Inhibition Rate (%) = (Control Area - Treatment Area)/Control Area × 100

#### 2.2.3 Spore germination inhibition assay

One milliliter of myxobacterial suspension (OD_600_ = 2.0) was mixed with 1 mL of fungal spore suspension (1.0 × 10^6^ spores/mL) and incubated at 28 °C and 180 rpm for 4 h. Spore germination was observed using a hemocytometer (100 spores per sample). A mixture of LBS broth and spore suspension served as the control. Each treatment was performed in triplicate, and the experiment was repeated twice independently. Germination was defined as the germ tube length exceeding half the spore length. Spore germination rate (%) and inhibition rate (%) were calculated according to Xue et al. (2013).

### 2.3 Effects of cell-free filtrates and volatile organic compounds

To assess the impact of cell-free filtrates (CFF), myxobacterial fermentation broth was centrifuged (12,000 rpm, 15 min, 4 °C) and the supernatant was passed through a 0.22-μm microporous membrane. CFF was mixed with VY/2 medium (at ∼50 °C) to a final concentration of 40% (v/v) to prepare plates (Akpinar et al., 2021). Sterile LBS broth served as the control. Fungal plugs (5 mm) were inoculated in the center and incubated at 28 °C for 5 d to calculate mycelial inhibition. Spore germination inhibition and spore lysis rates were determined as described in section 2.2.3, replacing the cell suspension with CFF. Each treatment was performed in triplicate, and the experiment was repeated twice independently. The lysis rate (%) was calculated as:

Lysis Rate (%) = (Control Spore Count - Treatment Spore Count)/Control Spore Count × 100

The activity of VOCs was evaluated using the dual-plate method (Kaddes et al., 2019). A 100-μL aliquot of myxobacterial suspension was spread on a VY/2 plate and cultured for 3 d. A separate PDA plate was inoculated with a fungal plug. The two plates were then inverted over each other, sealed with Parafilm, and incubated at 28 °C for 3 days. A sterile VY/2 plate served as the control. Each treatment was performed in triplicate, and the experiment was repeated twice independently.

### 2.4 Greenhouse pot trials

#### 2.4.1 Preparation of myxobacterial and pathogenic suspensions

Myxobacterial strains were inoculated (3% v/v) into 200 mL of LBS broth in 500-mL Erlenmeyer flasks containing 200 glass beads (3 mm diameter) to promote dispersed growth. Following incubation at 30 °C and 180 rpm for 3 days, the fermentation broth was adjusted with sterile water to an OD_600_ of 2.0 for further use. Solid inoculants were prepared following previously established protocols (Han et al., 2025). Briefly, *Protaetia brevitarsis* frass (provided by Jinkunchong Biotechnology Co., Ltd., Xinjiang, China) and crushed wheat straw were mixed at a 3:1 (w/w) ratio to serve as the solid fermentation substrate. The substrate was sterilized at 121°C (0.1 MPa) for 30 min. Myxobacterial fermentation broth was inoculated into the substrate at a rate of 100 mL/kg, thoroughly mixed, and adjusted to a final moisture content of 60% using sterile water. Following incubation at 30°C for 6 days, aliquots of the inoculant were placed on VY/2 medium to verify myxospore viability and ensure the absence of contamination. To quantify myxospore density, 1 g of the inoculant was suspended in 9 mL of sterile water, subjected to ultrasonication (950 W, 30% output, 3 s on/10 s off) for 5 min, and incubated in a water bath at 50°C for 2 h. Serial dilutions were plated to determine colony-forming units (CFU), with the final myxospore concentration reaching 10^6^ CFU/g. Uniformity was confirmed via multi-point sampling. The final product was dried at 50°C to a moisture content of 20%. While the inoculants used in this study were freshly prepared, the formulation remains stable for up to 180 days when sealed in bags at room temperature.

For pathogenic fungi, 200 g of millet was boiled for 3 min, rapidly cooled with water, and air-dried at room temperature. The millet was then sterilized in 500-mL flasks and inoculated with five mycelial plugs (5 mm diameter) of *Fpg*, *Fg*, or *Fc*. After 7 d of incubation in the dark at 28 °C, the individual millet inocula were mixed in a 1:1:1 (w/w/w) ratio to create a complex pathogen inoculum (hereafter referred to as F3) (Zhou et al., 2019).

#### 2.4.2 Biocontrol evaluation of fermentation broth

Wheat seeds were surface sterilized with 2% sodium hypochlorite (3 min), washed with sterile water, immersed in 75% ethanol (2 min), and rinsed thoroughly. The growth medium consisted of unsterilized nutrient soil (coconut coir, peat, carbonized rice husk, and perlite) mixed with vermiculite (4:1 v/v) in pots (130 mm × 140 mm). Pathogen inoculation was performed concurrently with sowing by spreading 1 g of inoculum per pot on the soil surface, followed by covering with a 1-cm soil layer. After emergence, seedlings were thinned to 10 plants per pot.

At the one-leaf stage (7 days post-emergence), seedlings were treated with myxobacterial fermentation broth. Experimental groups included single infections (*Fpg*, *Fg*, or *Fc*) and the complex infection (F3), with four treatments per group: (1) Myxobacteria + Pathogen: 1 mL of fermentation broth applied to the stem base per plant. (2) Fungicide + Pathogen: 1 mL of 43% Tebuconazole (500-fold dilution) applied to the stem base per plant. (3) Control (CK): 1 mL of sterile LBS broth applied per plant. (4) Mock: No pathogen or biocontrol treatment. Each treatment consisted of four replicates (10 plants per pot), and the experiment was repeated three times.

To assess the effect of myxobacteria on wheat growth, a separate set of non-infected wheat was treated with fermentation broth, LBS broth, or water. Each treatment consisted of four replicates (10 plants per pot), and the experiment was repeated three times independently.

#### 2.4.3 Biocontrol evaluation of solid agents

Experimental groups and pathogen inoculation remained consistent with the liquid trials. The treatments were: (1) Solid Agent + Pathogen: 1 g of solid myxobacterial agent spread per pot before sowing, followed by 1 g of pathogen inoculum. (2) Sterile Carrier + Pathogen: Method as above, using sterile fermentation substrate without myxobacteria. (3) Fungicide + Pathogen: As described in section 2.5.2. (4) CK: Pathogen inoculation only (1 g/pot). Each treatment consisted of four replicates (10 plants per pot), and the experiment was repeated three times.

Growth promotion assays for solid agents were conducted similarly, using solid agent, sterile carrier, or untreated soil. Each treatment consisted of four replicates (10 plants per pot), and the experiment was repeated three times independently.

#### 2.4.4 Disease assessment and agronomic traits

Pots were arranged randomly in a greenhouse (25–28 °C, relative humidity > 60%). Disease incidence and severity were recorded 35 d post-inoculation. Severity was graded on a 7-level scale (0–7) as defined by (Zhou et al., 2019). The disease index (DI) and biocontrol efficacy were calculated as follows:


DI=Σ(No.ofdiseasedplantsateachlevel×Levelvalue)/

Total⁢plants×Maximum⁢level⁢value×100

Efficacy(%)=(DI-controlDI)treatment/DI×control100

Additionally, plant height, primary root length, and fresh weight were measured.

### 2.5 Field efficacy trials

Field experiments were conducted from April to July 2025 at the Emin Wheat Experimental Base, Xinjiang Academy of Agricultural Sciences (46°34’10.5″N, 83°43’27.4″E). The trial was conducted in a loamy soil (pH 7.8–8.2; organic matter 18.8 g/kg) containing 0.89 g/kg TN, 9.3 mg/kg available P, and 71.5 mg/kg available K. The preceding crop was maize, and the site was free of recent FCR outbreaks. Meteorological conditions from flowering to grain-filling (June–July) were characterized by mean daily temperatures of 20–28°C (max >30°C) and extremely low rainfall (3.8–5.7 mm/month). Due to minimal precipitation, water requirements were met primarily through drip irrigation.

Field trials were conducted using a Randomized Complete Block Design (RCBD). Each experimental plot measured 5 m × 1 m, with six rows per plot and a 15-cm row spacing. The trial comprised 10 treatments, each with three replicates, totaling 30 plots. Disease incidence, disease index (DI), and control efficacy were calculated based on the three plots per treatment. The specific experimental layout is detailed in Supplementary Table 1.

Treatments 1–3 consisted of myxobacterial fermentation broths (HM-E, KT23, and KE15), while Treatments 4–6 utilized the corresponding solid inoculants. Treatment 7 served as a sterile solid-substrate control, and Treatment 8 utilized Tebuconazole as a chemical control. Treatment 9 was the infected control (pathogen only), and Treatment 10 served as the blank control (no pathogen or myxobacteria). For Treatments 1–9, a composite pathogen inoculum was incorporated into the soil at a depth of 3–5 cm at a rate of 70 g/m^2^ prior to sowing.

Disease surveys were conducted at the flowering and grain-filling stages using 5-point diagonal sampling (40 plants per point). Severity was graded on a 5-level scale (0–5) according to the national standard NY/T 4179-2022. Agronomic parameters (plant height, stem length under spike, flag leaf area, and second leaf area) were measured at the grain-filling stage. Yield was calculated at harvest, and the yield increase rate was determined relative to the control.

### 2.6 Morphological observation

Strains KT23 and KE15 were cultured on VY/2 for 5 days. Colony and fruiting body morphology were observed using a stereo microscope (SM7, Motic China Group Co., Ltd.). Vegetative cells and myxospores were observed via bright-field microscopy (Nikon Ni-U) after Gram staining and via scanning electron microscopy (SEM, Zeiss SUPRA55VP) at the Experimental Center of the Xinjiang Institute of Ecology and Geography, Chinese Academy of Sciences.

### 2.7 Molecular identification

Genomic DNA was extracted using a TIANGEN kit. The 16S rRNA gene was amplified with primers 27F/1492R (Stackebrandt et al., 2005). Additionally, partial sequences of the *lepA* gene (leader peptidase GTP binding membrane protein) were amplified with BAUP1/BIDN1 (Kumar et al., 2017), and *gyrB* (DNA gyrase subunit B) with gyrBF/gyrBR (Santos and Ochman, 2004).

PCR products were sequenced (Sangon Biotech, Shanghai) and aligned using BLAST against the NCBI database. Phylogenetic trees were constructed using the neighbor-joining method in MEGA 11.0.

### 2.8 Data analysis

For *in vitro* assays (e.g., plate confrontation and spore germination), three replicates were used per treatment, and experiments were repeated twice; results are presented for one representative experiment (*n* = 3). For greenhouse pot trials, four pots (10 plants/pot) were used per treatment across three independent repetitions (*n* = 4). Field trial data were analyzed based on three plots per treatment (*n* = 3). Data were analyzed using one-way analysis of variance (ANOVA) followed by Duncan’s multiple range test to determine significant differences between treatments. Statistical analyses were performed using SPSS 19.0 software (SPSS Inc., Chicago, IL, United States). A *P* < 0.05 was considered statistically significant. All data visualization and graphical representations were generated using GraphPad Prism 8.0.

## 3 Results

### 3.1 Growth inhibition of *Fpg*, *Fg*, and *Fc* by myxobacteria

**FIGURE 1:**
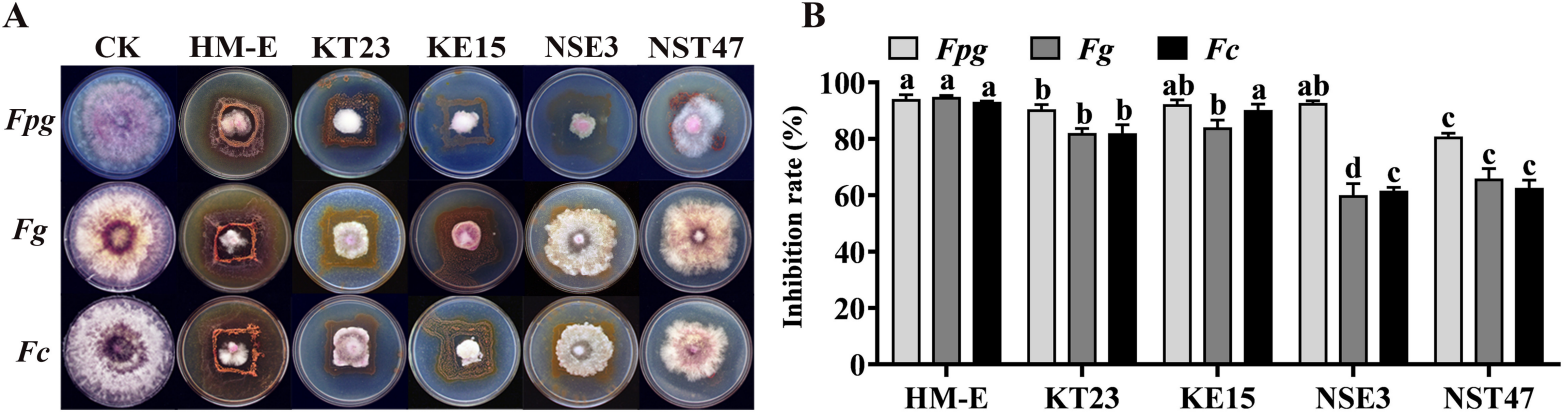
Antagonistic activity of selected myxobacteria against Fusarium pathogens. **(A)** Antifungal activity of selected myxobacterial strains against *Fusarium pseudograminearum* (*Fpg*), *Fusarium graminearum* (*Fg*), and *Fusarium culmorum* (*Fc*) following 5 days of co-cultivation on VY/2 agar. **(B)** Inhibition rates of myxobacteria against *Fpg*, *Fg*, and *Fc*. Data are expressed as means ± standard deviation (SD). For each treatment, three plates were included, and the experiment was repeated twice. The results shown are derived from a single representative experiment (*n* = 3). Different letters indicate significant differences (*P* < 0.05) according to Duncan’s multiple range test.

Initial screening against *F. pseudograminearum* (*Fpg*) via dual-culture assays revealed that 73 out of 81 tested myxobacterial strains exhibited varying degrees of antagonistic activity (Supplementary Table 2). Five strains (HM-E, KT23, KE15, NSE3, and NST47) showed inhibition rates exceeding 85.56%. Further evaluation of these five strains against *Fpg*, *Fg*, and *Fc* demonstrated broad-spectrum antifungal activity. Notably, the myxobacteria were observed to spread along the fungal hyphae, causing them to become sparse and collapse, eventually occupying a large surface area—a clear indication of predatory behavior (Figure 1A). Among these, strains HM-E, KT23, and KE15 exhibited the strongest antagonistic potential, with inhibition rates against *Fpg*, *Fg*, and *Fc* ranging from 82.01 to 94.84% (Figure 1B).

### 3.2 Effects of myxobacteria on spore germination

**FIGURE 2:**
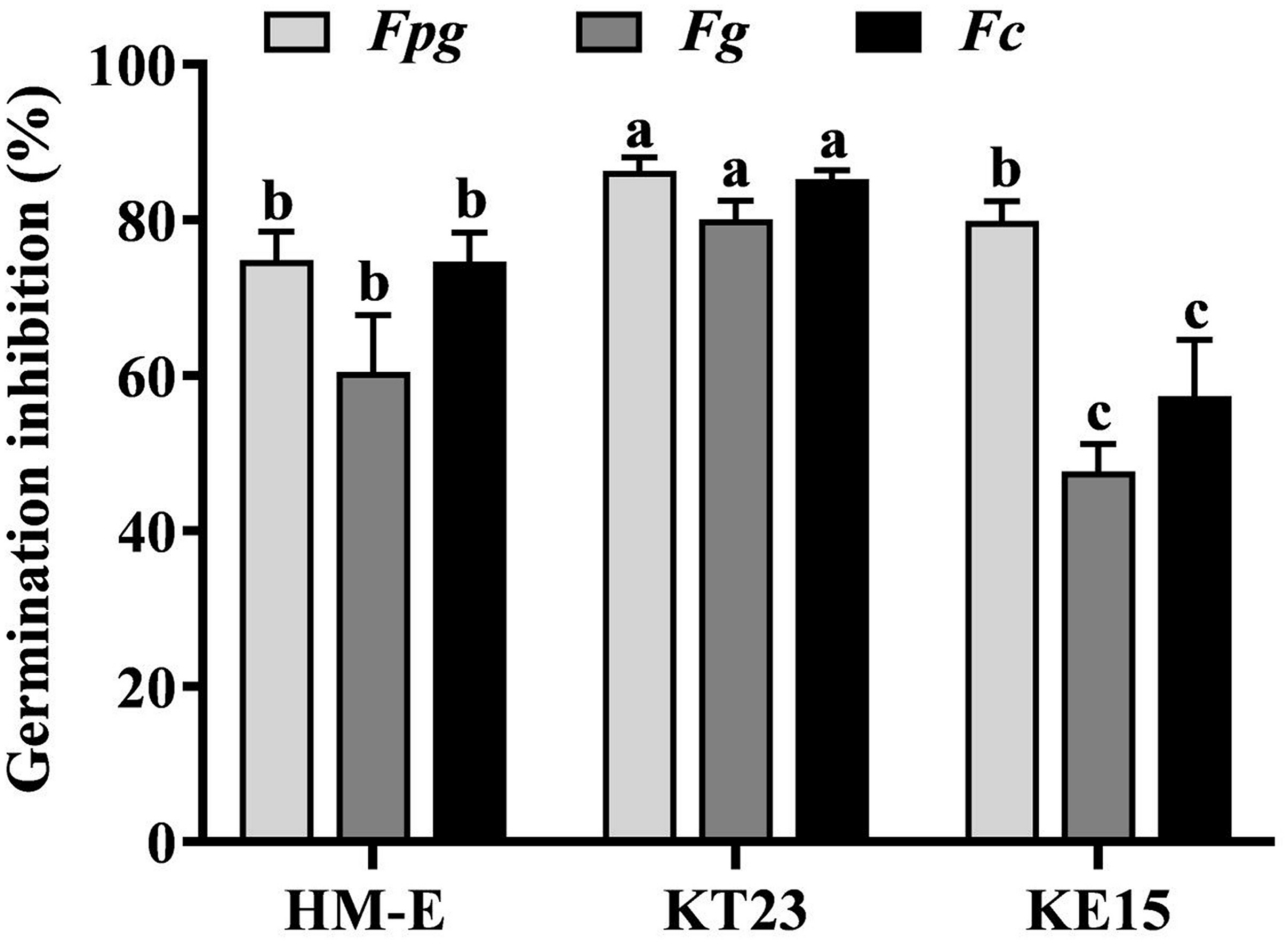
Inhibition of spore germination of Fusarium pathogens by three myxobacterial strains. Spore germination inhibition rates of *Fpg*, *Fg*, and *Fc* treated with three myxobacterial strains. Data are expressed as means ± standard deviation (SD). For each treatment, three replicates were included, and the experiment was repeated twice. The results shown are derived from a single representative experiment (*n* = 3). Different letters indicate significant differences (*P* < 0.05) according to Duncan’s multiple range test.

Based on the dual-culture results, strains HM-E, KT23, and KE15 were selected to evaluate their inhibitory effects on the spore germination of *Fpg*, *Fg*, and *Fc*. As shown in Figure 2, all three strains significantly inhibited germination and induced spore lysis. Specifically, the germination inhibition rates for HM-E, KT23, and KE15 were 74.84, 86.34, and 79.88% for *Fpg*; 60.45, 80.07, and 47.64% for *Fg*; and 74.66, 85.27, and 57.35% for *Fc*, respectively.

### 3.3 Antifungal activity of cell-free filtrates and volatile organic compounds

**FIGURE 3:**
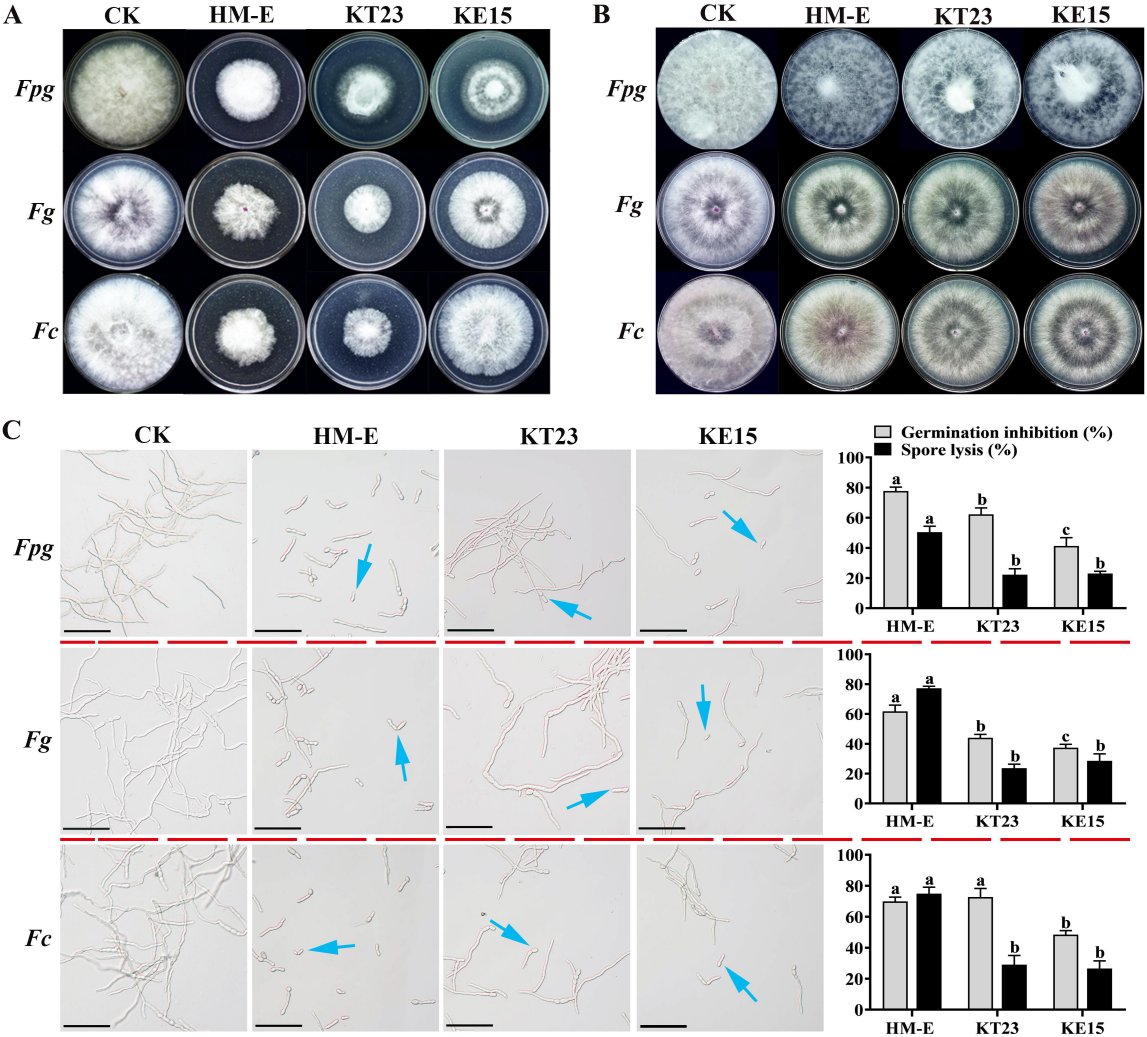
Effects of myxobacterial cell-free fermentation filtrates and volatile organic compounds on Fusarium growth and spores. **(A)** Inhibitory effects of cell-free fermentation filtrates on mycelial growth of *Fpg*, *Fg*, and *Fc*. **(B)** Inhibitory effects of myxobacterial volatile organic compounds (VOCs) on mycelial growth of *Fpg*, *Fg*, and *Fc*. **(C)** Effects of cell-free fermentation filtrates on spore lysis and germination of *Fpg, Fg*, and *Fc* (blue arrows indicate inhibited spore germination). Scale bar = 10 μm. Data are expressed as means ± standard deviation (SD). For each treatment, three plates were included, and the experiment was repeated twice. The results shown are derived from a single representative experiment (*n* = 3). Different letters indicate significant differences (*P* < 0.05) according to Duncan’s multiple range test.

Compared to the control, the cell-free filtrates (CFF) of strains HM-E, KT23, and KE15 significantly inhibited the mycelial growth of the three pathogens (Figure 3A). Furthermore, the CFF of all three strains induced spore lysis and inhibited germination (Figure 3C). For *Fpg*, the germination inhibition rates were 77.76, 62.31, and 41.28%, with lysis rates of 50.47, 22.28, and 22.99%. For *Fg*, germination inhibition reached 61.79, 44.09, and 37.39%, with lysis rates of 77.23, 23.66, and 28.57%. For *Fc*, the rates were 69.94, 72.73, and 48.44% for germination inhibition, and 74.92, 29.05, and 26.61% for lysis.

Regarding VOCs, strains HM-E, KT23, and KE15 all inhibited the mycelial growth of *Fpg*, *Fg*, and *Fc*. While the aerial hyphae became noticeably sparser following treatment compared to the control, the inhibitory effect was not statistically significant (Figure 3B).

### 3.4 Greenhouse biocontrol efficacy and growth promotion

#### 3.4.1 Efficacy of fermentation broth

**FIGURE 4:**
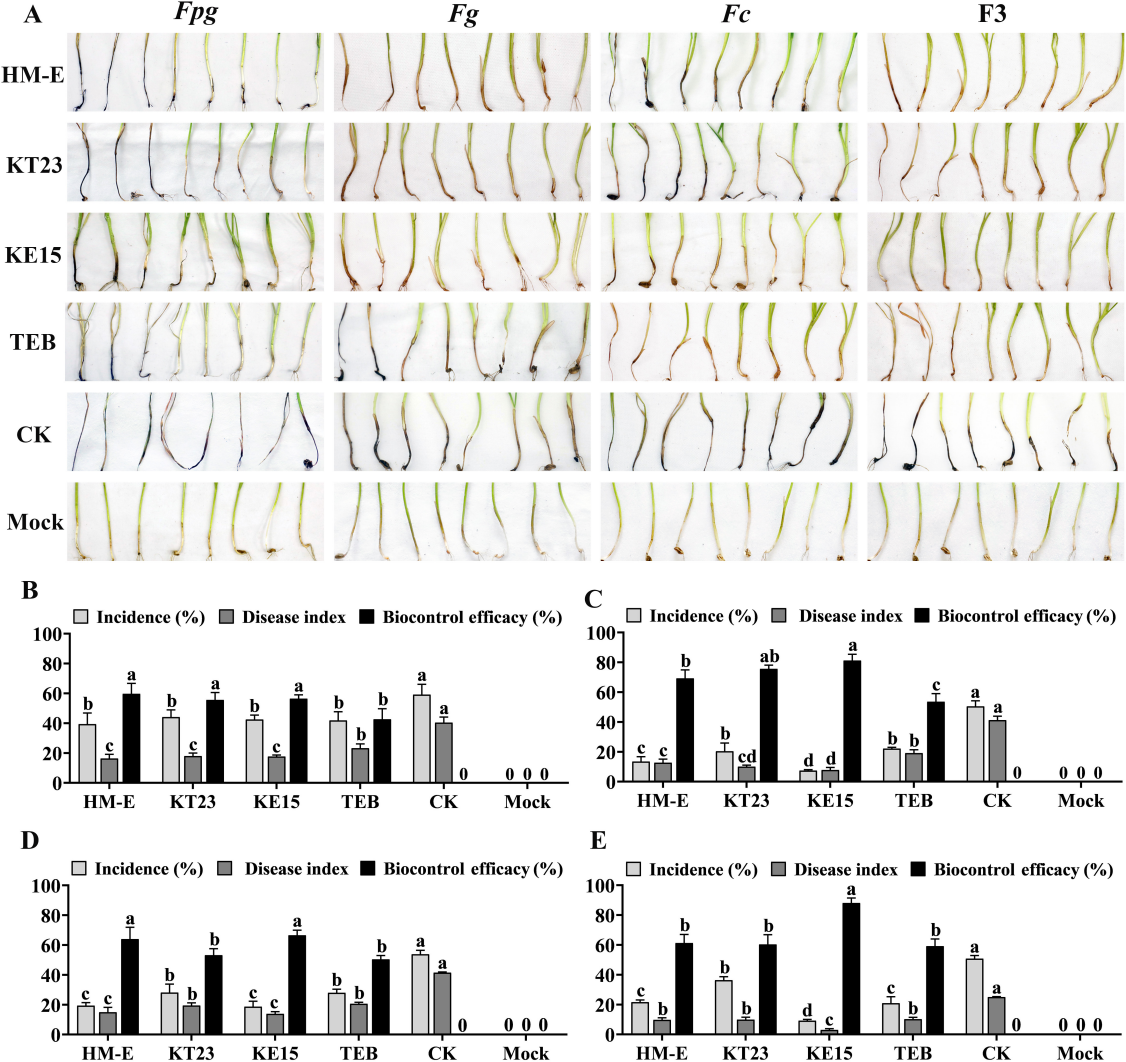
Biocontrol efficacy of myxobacterial fermentation filtrates against wheat crown rot under greenhouse conditions. Biocontrol efficacy of fermentation filtrates from myxobacterial strains HM-E, KT23, and KE15 against wheat crown rot caused by single or mixed infections of *Fusarium* spp. under greenhouse conditions. **(A)** Disease suppression effects against wheat crown rot caused by single infections of *Fpg*, *Fg*, or *Fc*, and mixed infection with all three pathogens (F3). TEB represents the chemical control treated with tebuconazole; CK represents the pathogen-inoculated control; Mock represents healthy wheat plants without pathogen inoculation. **(B–D)** Effects of myxobacterial fermentation filtrates on disease incidence, disease index, and control efficacy in wheat infected with *Fpg* **(B)**, *Fg* **(C)**, and *Fc* **(D)**, respectively. **(E)** Effects of myxobacterial fermentation filtrates on disease incidence, disease index, and control efficacy in wheat co-infected with *Fpg*, *Fg*, and *Fc* (F3). Data are expressed as means ± SD. Each treatment consisted of four pots with 10 plants per pot. The experiment was conducted three times; results are presented from a single representative experiment (*n* = 4 replicates, 40 plants total). Different letters indicate significant differences (*P* < 0.05) based on Duncan’s multiple range test.

Inoculation with myxobacterial fermentation broth significantly reduced both the disease incidence and disease index (DI) of FCR caused by single or complex infections (*P* < 0.05; Figure 4). The biocontrol efficacies of HM-E, KT23, and KE15 were 59.69, 55.55, and 56.41% against *Fpg* (Figures 4A,B); 69.20, 75.64, and 81.16% against *Fg* (Figures 4A,C); and 64.00, 53.15, and 66.56% against *Fc* (Figures 4A,D). Against the F3 complex infection, the efficacies were 61.23, 60.33, and 88.11%, respectively (Figures 4A,E). In all groups, the fermentation broth performed better than 43% Tebuconazole.

#### 3.4.2 Efficacy of solid agents

**FIGURE 5:**
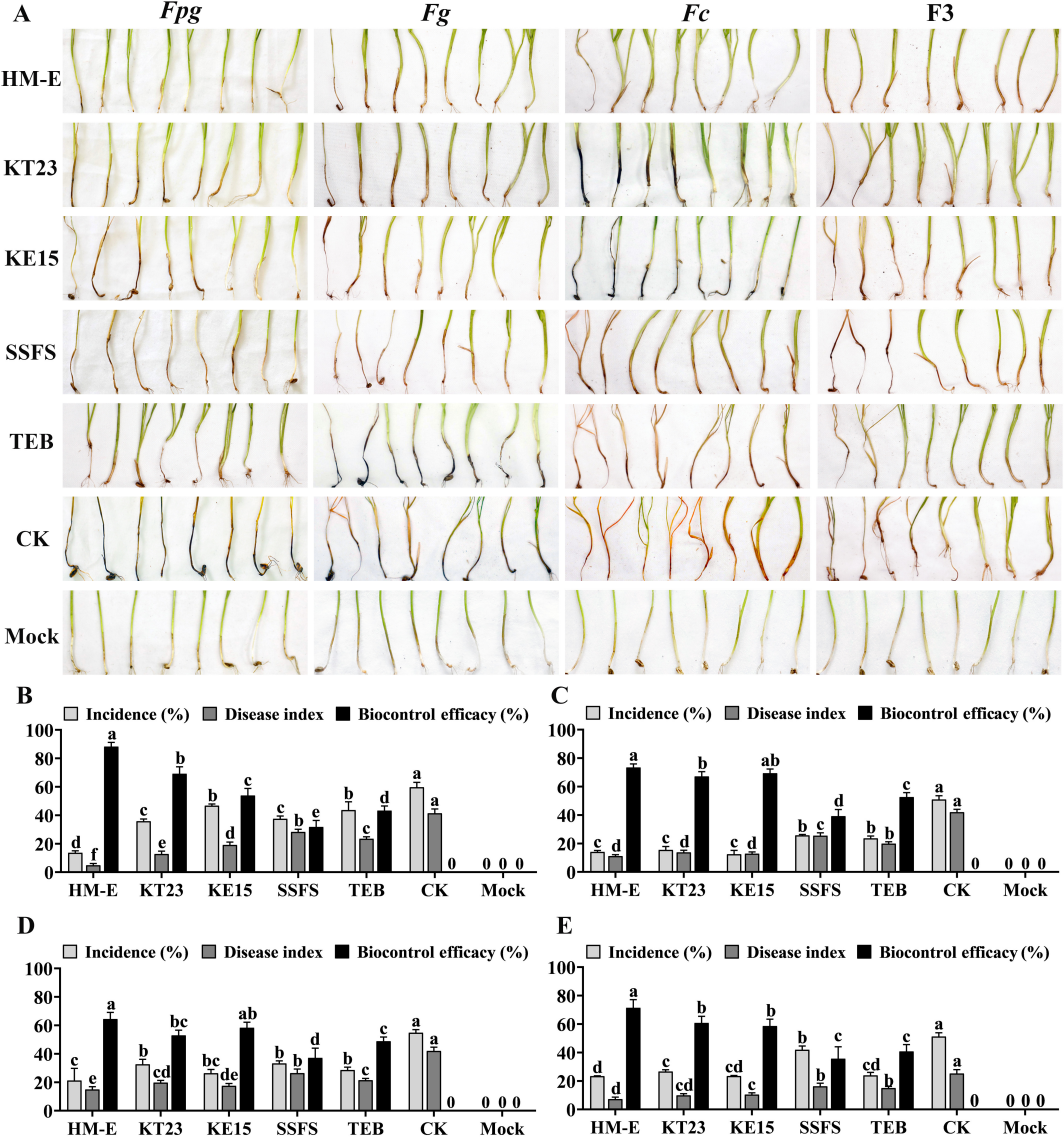
Biocontrol efficacy of myxobacterial solid inoculants against wheat crown rot under greenhouse conditions. **(A)** Disease suppression by solid inoculants of HM-E, KT23, and KE15 against wheat crown rot caused by single infections of *Fusarium pseudograminearum* (*Fpg*), *Fusarium graminearum* (*Fg*), *Fusarium culmorum* (*Fc*), and mixed infection with all three pathogens (F3). SSFS represents the sterile solid fermentation substrate; TEB represents the chemical control treated with tebuconazole; CK represents the pathogen-inoculated control; Mock represents healthy wheat plants without pathogen inoculation. **(B–D)** Effects of myxobacterial solid inoculants on disease incidence, disease index, and control efficacy in wheat infected with *Fpg* **(B)**, *Fg* **(C)**, and *Fc* **(D)**, respectively. **(E)** Effects of myxobacterial solid inoculants on disease incidence, disease index, and control efficacy in wheat under mixed infection with *Fpg*, *Fg*, and *Fc* (F3). Data are expressed as means ± SD. Each treatment consisted of four pots with 10 plants per pot. The experiment was conducted three times; results are presented from a single representative experiment (*n* = 4 replicates, 40 plants total). Different letters indicate significant differences (*P* < 0.05) based on Duncan’s multiple range test.

Similarly, the solid agents of HM-E, KT23, and KE15 significantly reduced FCR severity across all infection types (*P* < 0.05; Figure 5). Biocontrol efficacies against *Fpg* were 88.24, 69.17, and 53.87% for the three strains, while the sterile carrier alone provided 31.76% efficacy (Figures 5A,B). Against *Fg*, efficacies were 73.46, 67.14, and 69.43% (carrier: 39.23%; Figures 5A,C). Against *Fc*, the rates were 64.41, 52.94, and 58.32% (carrier: 37.14%; Figures 5A,D). For the F3 complex infection, efficacies were 71.42, 60.82, and 58.64% (carrier: 35.64%; Figures 5A,E). The solid agents consistently outperformed Tebuconazole.

#### 3.4.3 Effects on wheat growth

**FIGURE 6:**
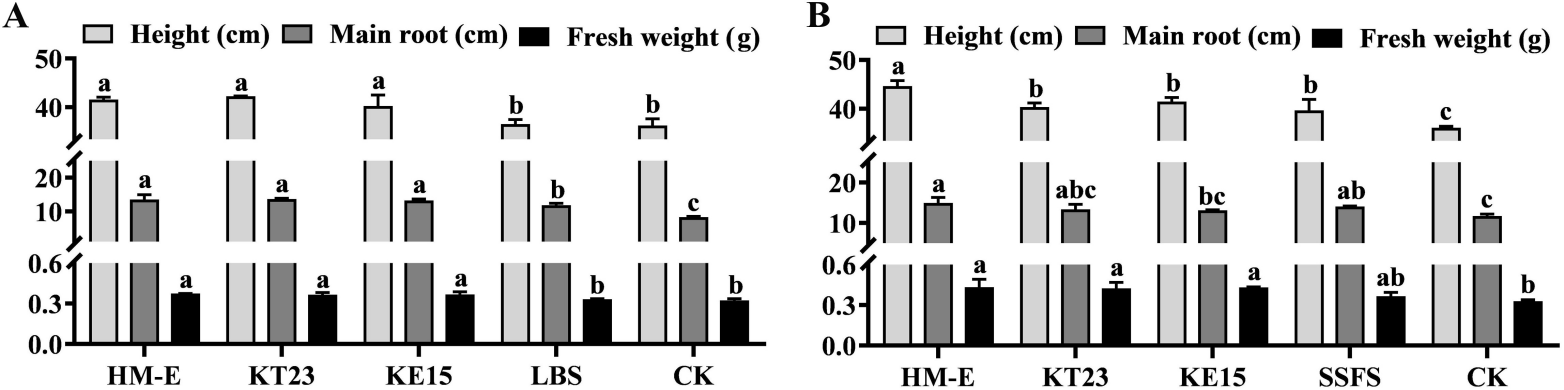
Effects of myxobacteria HM-E, KT23, and KE15 on wheat growth. **(A)** Effects of myxobacterial fermentation broths on wheat plant height, primary root length, and fresh weight. LBS represents the sterile LBS medium control. **(B)** Effects of myxobacterial solid inoculants on wheat plant height, primary root length, and fresh weight. SSFS represents the sterile solid fermentation substrate control. Data are expressed as means ± SD. Each treatment consisted of four pots with 10 plants per pot. The experiment was conducted three times; results are presented from a single representative experiment (*n* = 4 replicates, 40 plants total). Different letters indicate significant differences (*P* < 0.05) based on Duncan’s multiple range test.

In the absence of pathogens, all three myxobacterial fermentation broths significantly promoted wheat growth; plant height, primary root length, and total fresh weight were significantly higher than both the LBS and Mock controls (Figure 6A). In the solid agent groups, all three strains significantly increased plant height and fresh weight compared to the Mock control. However, compared to the sterile carrier treatment, only strain HM-E significantly increased plant height, while strains KT23 and KE15 showed no significant difference (Figure 6B).

### 3.5 Field biocontrol efficacy

Field results indicated that both fermentation broths and solid agents significantly reduced the disease incidence and DI compared to the positive control (*P* < 0.05; Table 1). At the flowering stage, the efficacy of fermentation broths ranged from 61.60 to 70.67%, and solid agents ranged from 63.20 to 74.67% (sterile carrier: 38.67%). By the grain-filling stage, fermentation broth efficacy was 61.94–71.53% and solid agents were 57.95–63.74% (sterile carrier: 32.23%). Both myxobacterial treatments outperformed 43% Tebuconazole (efficacy: 52.27% at flowering and 53.36% at grain-filling).

**TABLE 1:** Field control efficacy of myxobacterial treatments against wheat crown rot.

No.	Treatment	Flowering stage			Grain-filling stage		
		Disease incidence (%)	Disease index	Efficacy (%)	Disease incidence (%)	Disease index	Efficacy (%)
1	HM-E fermentation broth	16.83 ± 0.44 bc	4.10 ± 0.35 cd	67.20 ± 2.81 ab	21.83 ± 0.73 g	10.00 ± 0.29 d	71.53 ± 0.82 a
2	KE15 fermentation broth	18.00 ± 1.32 bc	4.80 ± 0.79 cd	61.60 ± 5.06 ab	29.33 ± 0.93 f	11.60 ± 1.25 cd	66.97 ± 3.57 ab
3	KE15 solid inoculant	17.50 ± 1.53 bc	4.60 ± 0.83 cd	63.20 ± 6.66 ab	34.50 ± 2.93 def	12.73 ± 0.35 cd	63.74 ± 0.99 abc
4	KT23 fermentation broth	14.67 ± 1.09 c	3.67 ± 0.64 cd	70.67 ± 5.09 ab	30.50 ± 0.50 ef	13.37 ± 0.69 cd	61.94 ± 1.96 abc
5	HM-E solid inoculant	18.00 ± 5.01 bc	4.13 ± 1.09 cd	66.93 ± 4.06 ab	38.83 ± 1.59 cd	14.37 ± 1.07 cd	59.09 ± 3.04 abc
6	KT23 solid inoculant	13.50 ± 1.04 c	3.17 ± 0.26 d	74.67 ± 2.08 a	37.17 ± 1.69 cde	14.77 ± 0.81 cd	57.95 ± 2.30 bc
7	Tebuconazole (Chemical control)	20.33 ± 1.01 bc	5.97 ± 0.23 bc	52.27 ± 1.87 bc	56.71 ± 3.52 b	16.38 ± 1.34 c	53.36 ± 3.83 c
8	Sterile solid fermentation substrate (control)	23.83 ± 2.89 b	7.67 ± 1.20 b	38.67 ± 9.60 c	43.17 ± 1.69 c	23.80 ± 3.22 b	32.23 ± 9.18 d
9	CK	62.50 ± 0.64 a	12.50 ± 0.76 a	–	75.19 ± 2.28 a	35.12 ± 0.83 a	–
10	Mock	–	–	–	–	–	–

Data represent means ± SD (*n* = 3). Three plots were assigned per treatment, with 200 wheat plants investigated per plot. Results are based on a single field trial. Different letters within a column indicate significant differences (*P* < 0.05) according to Duncan’s multiple range test.

Agronomic surveys at the grain-filling stage showed that myxobacterial treatments significantly mitigated the growth inhibition caused by the disease (Table 2). At harvest, the yield for the positive control was 5,860.00 kg/ha. In contrast, fermentation broth treatments yielded 6,663.33–7,096.67 kg/ha (a 14.90–21.10% increase over the positive control), and solid agents reached 7,400.00–7,490.00 kg/ha (a 26.28–27.82% increase). Notably, the solid agents increased yield by 2.49–3.74% even when compared to the healthy Mock control.

**TABLE 2:** Effects of myxobacterial treatments on wheat growth and yield under field conditions.

Treatment	Plant height (cm)	Peduncle length (cm)	Flag leaf area (cm^2^)	Last second leaf area (cm^2^)	Yield (Kg/ha)	Yield increase vs. CK (%)	Yield increase vs. Mock (%)
HM-E fermentation broth	77.01 ± 0.55 bc	31.80 ± 0.23 c	25.03 ± 2.42 a	21.01 ± 4.23 ab	7096.67 ± 136.91 cd	21.10 ± 2.34 b	–1.71 ± 1.90 b
KT23 fermentation broth	78.56 ± 1.85 ab	34.03 ± 0.36 bc	21.43 ± 1.72 ab	18.36 ± 1.72 ab	6663.33 ± 61.73 f	13.71 ± 1.05 d	–7.71 ± 0.86 d
KE15 fermentation broth	78.55 ± 1.96 ab	32.98 ± 0.71 c	24.27 ± 1.85 a	21.52 ± 1.61 ab	6733.33 ± 54.67 ef	14.90 ± 0.93 cd	–6.74 ± 0.76 cd
HM-E solid inoculant	82.53 ± 1.12 ab	33.21 ± 0.60 bc	25.61 ± 1.74 a	19.43 ± 2.04 ab	7490.00 ± 51.96 a	27.82 ± 0.89 a	3.74 ± 0.72 a
KT23 solid inoculant	84.64 ± 0.67 a	35.38 ± 0.14 ab	26.33 ± 1.43 a	21.97 ± 0.42 a	7480.00 ± 70.24 a	27.65 ± 1.20 a	3.60 ± 0.97 a
KE15 solid inoculant	79.23 ± 4.75 ab	33.73 ± 1.44 bc	25.38 ± 1.35 a	21.03 ± 2.46 ab	7400.00 ± 40.00 ab	26.28 ± 0.68 a	2.49 ± 0.55 a
Sterile solid fermentation substrate (control)	79.76 ± 3.10 ab	32.70 ± 0.92 c	22.42 ± 0.29 ab	19.01 ± 2.62 ab	7110.00 ± 40.41 cd	21.33 ± 0.69 b	–1.52 ± 0.56 b
TEB	81.91 ± 0.41 ab	36.56 ± 0.06 a	23.12 ± 0.05 a	18.86 ± 0.26 ab	6906.67 ± 17.64 de	17.86 ± 0.30 bc	–4.34 ± 0.24 bc
CK	69.78 ± 1.56 c	32.19 ± 0.63 c	17.52 ± 0.57 b	14.85 ± 0.97 b	5860.00 ± 64.29 g	–	-18.84 ± 0.89 e
Mock	80.27 ± 0.58 ab	33.66 ± 0.38 bc	25.24 ± 0.79 a	20.11 ± 0.17 ab	7220.00 ± 80.83 bc	–	–

Data represent means ± SD (*n* = 3). Three plots were assigned per treatment, with 40 plants per plot evaluated for agronomic traits. Results are based on three independent replicates from a single field trial. Different letters within a column indicate significant differences (*P <* 0.05) according to Duncan’s multiple range test. The color shaded rows represent the three bacterial solid agents in order from highest performance to lowest. The others without color shaded are controls.

### 3.6 Myxobacterial morphology

**FIGURE 7:**
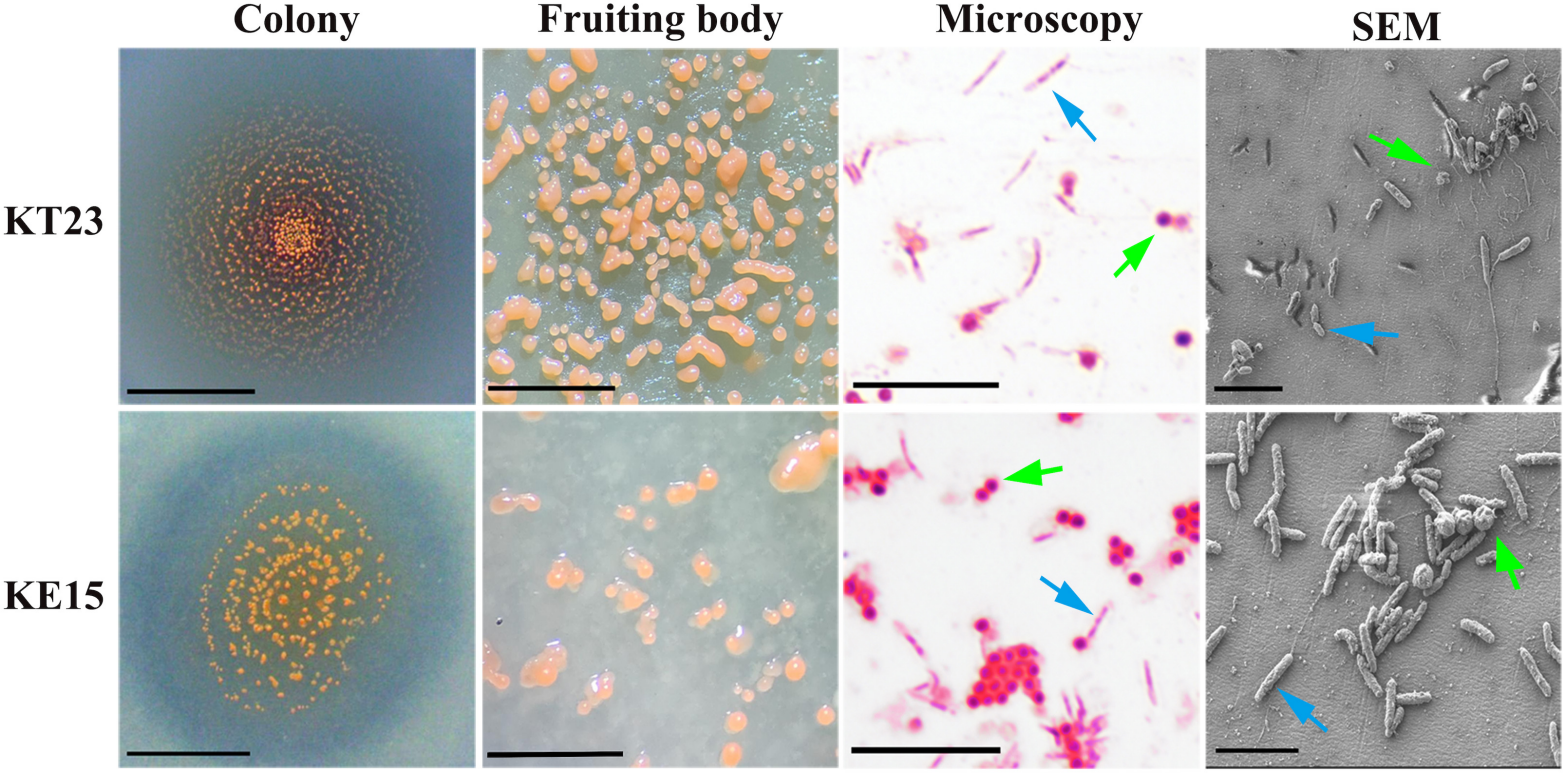
Morphological characteristics of myxobacterial strains KT23, and KE15. Colony morphology (scale bar = 3 mm); fruiting body formation (scale bar = 30 μm); light microscopy showing vegetative cells (blue arrows) and myxospores (green arrows) (scale bar = 15 μm); and scanning electron microscopy (SEM) images (scale bar = 5 μm).

Strains KT23 and KE15 exhibited similar morphological characteristics. On VY/2 plates, the biofilms expanded as thin films, eventually forming numerous orange spherical fruiting bodies. The fruiting bodies were solitary; vegetative cells were rod-shaped (2–10 μm long), and myxospores were spherical (Figure 7).

### 3.7 Molecular identification

**FIGURE 8:**
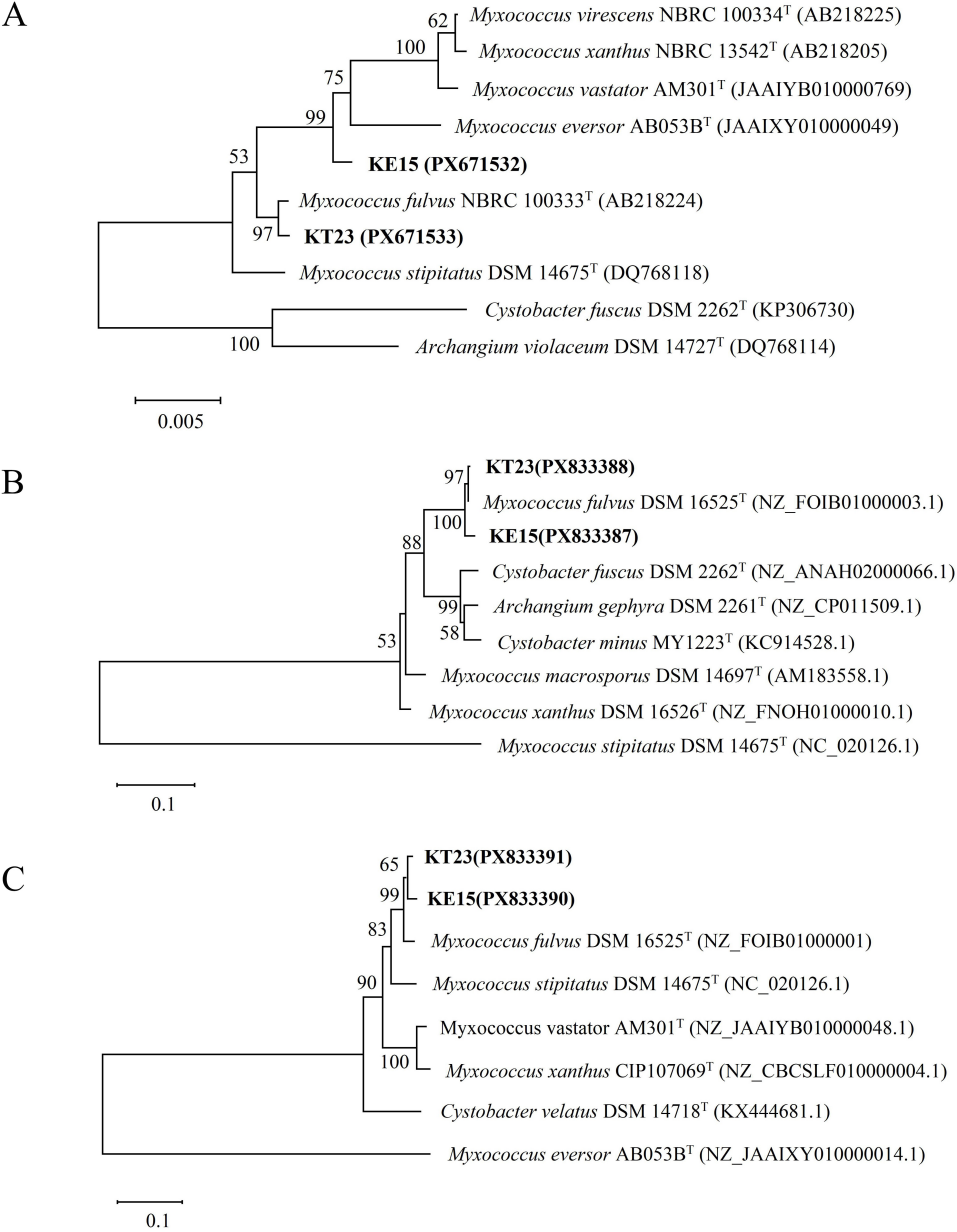
Phylogenetic analysis of myxobacterial strains KT23, and KE15. Molecular identification of myxobacterial strains KT23, and KE15 based on phylogenetic analysis of 16S rRNA, *lepA*, and *gyrB* gene sequences. Phylogenetic trees were constructed using the Neighbor-Joining method in MEGA 11.0. **(A)** Phylogenetic tree based on 16S rRNA gene sequences. **(B)** Phylogenetic tree based on *lepA* gene sequences. **(C)** Phylogenetic tree based on *gyrB* gene sequences. Bootstrap values (percentage of 1,000 replicates) are shown at branch nodes.

PCR amplification and sequencing of 16S rRNA, *lepA*, and *gyrB* genes were performed. The GenBank accession numbers are as follows: 16S rRNA (PX671533, PX671532), *lepA* (PX833388, PX833387), and *gyrB* (PX833391, PX833390). In the 16S rRNA phylogenetic tree, strains KT23 and KE15 clustered with *Myxococcus* sp. type strains (Figure 8A). Multigene analysis using *lepA* and *gyrB* placed both strains in a single clade with *Myxococcus fulvus* DSM 16525^T^ (Figures 8B,C). Based on morphological features and multigene sequence analysis, KT23 and KE15 were identified as *Myxococcus fulvus*.

## 4 Discussion

The application of myxobacteria in plant disease control is a relatively recent but promising field, offering unique advantages due to their sophisticated biological traits. Unlike traditional biocontrol agents that rely solely on antibiotic production, myxobacteria utilize a “wolf-pack” predatory strategy, allowing them to actively lyse a wide range of pathogenic microorganisms (Muñoz-Dorado et al., 2016). This predatory lifestyle reduces the likelihood of pathogens developing resistance and enhances long-term ecological safety. Furthermore, myxobacteria are prolific producers of over 500 structurally novel secondary metabolites with potent antimicrobial activities, serving as a vast reservoir for lead compound discovery (Hyun and Cho, 2018). Additionally, their ability to differentiate into stress-resistant myxospores ensures survival under adverse conditions, such as drought or high temperatures (Lall et al., 2024). In this study, we identified three strains—HM-E, KT23, and KE15—with potent antagonistic activity against FCR pathogens, significantly expanding the known biocontrol repertoire of myxobacteria.

Our study demonstrates that the inhibitory effects of these bacterial strains against *Fusarium*—the primary causal agent of wheat crown rot (FCR)—involve a multi-faceted strategy encompassing both direct predation and indirect antibiosis. These two mechanisms are not mutually exclusive but rather function synergistically. In plate confrontation assays, we observed the directional expansion of myxobacterial colonies toward fungal hyphae, culminating in the structural collapse of the fungal colonies. This behavior aligns with the “wolf-pack” social predation model, in which large populations of myxobacterial cells move coordinately to surround and lyse prey (Muñoz-Dorado et al., 2016), actively seeking out and eliminating pathogens (Radford and Whitworth, 2024). This predatory capability provides a distinct ecological advantage over conventional biocontrol agents, which typically rely solely on the passive secretion of antimicrobial metabolites. Furthermore, the cell-free filtrates (CFF) of strains HM-E, KT23, and KE15 exhibited significant antifungal activity against all three *Fusarium* species tested, confirming the presence of potent secreted inhibitory factors.

In fact, an intrinsic link exists between the predatory and antibiotic mechanisms in myxobacteria. During the predatory process, myxobacteria secrete a diverse repertoire of hydrolases—including proteases, glycosyl hydrolases, and chitinases—alongside antibiotic secondary metabolites (Radford and Whitworth, 2024; Li et al., 2019; Li et al., 2023). These substances serve as “molecular weapons” designed to degrade prey cell walls and membranes, while simultaneously possessing independent, broad-spectrum antimicrobial properties (Xiao et al., 2011; Bhat et al., 2021). Furthermore, recent findings have revealed that myxobacteria can release outer membrane vesicles (OMVs) to mediate complex interactions with fungi, further elucidating the sophisticated synergy between predation and antibiosis (Wang et al., 2025). Consequently, we propose that the biocontrol efficacy of myxobacteria against FCR pathogens is the integrated result of direct predation and antibiotic action. This multi-pronged mechanism likely reduces the evolutionary pressure on pathogens to develop resistance, offering a more sustainable approach to disease management (Nair et al., 2019).

Microbial volatile organic compounds (VOCs) are low-molecular-weight molecules that diffuse easily through soil and air to inhibit pathogens (Schulz-Bohm et al., 2017). We observed that VOCs from our strains induced thinning of aerial hyphae in *Fusarium* species, although the inhibition was less pronounced than that of liquid metabolites. This is consistent with findings in *Corallococcus* sp. EGB, where VOCs such as 2-ethyl-1-hexanol were identified as key antifungal components capable of disrupting cell membrane integrity (Ye et al., 2020a).

However, the practical efficacy of volatile organic compounds (VOCs) in wheat fields is subject to complex environmental constraints, including soil aeration, temperature fluctuations, moisture levels, and the adsorptive capacity of soil particles. Consequently, the actual concentration and effective diffusion range of VOCs under field conditions are likely substantially attenuated compared to those observed in controlled *in vitro* assays. Therefore, the relative contribution of VOCs to the overall biocontrol efficacy in the field may be less pronounced than laboratory results suggest. Nevertheless, as highlighted by Schulz-Bohm et al. (2017), microbial VOCs can still reach biologically active concentrations within localized soil micro-environments, such as rhizosphere micropores and internal biofilm structures. Whether myxobacteria-derived VOCs exert a significant auxiliary inhibitory effect in the soil remains to be validated through *in situ* field detection techniques, such as solid-phase microextraction coupled with gas chromatography–mass spectrometry (SPME-GC-MS).

The transition from lab to field is the ultimate test for any biocontrol agent. Our trials demonstrated that both fermentation broths and solid agents significantly reduced FCR incidence and severity. Notably, field efficacies at the flowering (61.60–74.67%) and grain-filling stages (57.95–71.53%) consistently outperformed the commercial fungicide Tebuconazole. However, since these trials were conducted in the arid climate of Xinjiang, the stability and adaptability of these agents in other ecological regions with different soil types and indigenous microbial communities remain to be verified (Compant et al., 2019).

Beyond disease suppression, our myxobacterial treatments significantly enhanced wheat growth and yield. The yield increases in the field (13.71–21.10% for liquid; 26.28–27.82% for solid) were remarkable. Interestingly, solid agents increased yield by 2.49–3.74% even compared to healthy (non-inoculated) controls, confirming the growth-promoting effects observed in our greenhouse trials. In contrast to well-characterized plant growth-promoting rhizobacteria (PGPR) such as *Bacillus*, *Pseudomonas*, and *Azospirillum* (Santoyo et al., 2021), the capacity for indole-3-acetic acid (IAA) synthesis in myxobacteria has not been systematically investigated. Our laboratory’s prior evaluations of strains HM-E, KT23, and KE15 found no evidence of IAA production. Taken together, we hypothesize that the growth-promoting effects of myxobacteria likely stem from the following pathways: (1) Nutrient Mineralization: As predatory bacteria capable of degrading complex organic matter and nitrogenous compounds, myxobacteria may facilitate the release of nutrients from previously unavailable states, thereby enhancing soil nutrient bioavailability (Wang et al., 2020; Dai et al., 2025). (2) Rhizosphere Microbiome Modulation: Myxobacteria may optimize the structure of the rhizosphere microbial community by increasing the abundance of beneficial taxa while suppressing pathogenic populations. This shift in community composition creates a more resilient and healthier rhizosphere environment, which indirectly supports vigorous plant development (Ye et al., 2020b). While our current study confirms the growth-promoting potential of these myxobacterial strains in wheat, the precise mechanistic underpinnings and biochemical basis of this phenomenon require further elucidation through integrated multi-omic approaches, such as metagenomics and metabolomics.

Despite this potential, challenges remain for large-scale application. The relatively slow growth rate of myxobacteria necessitates further optimization of fermentation processes to improve efficiency and reduce costs (Keane and Berleman, 2016). Future research integrating metagenomics and metabolomics is required to fully elucidate their colonization patterns and multi-trophic interactions within the complex rhizosphere micro-ecosystem.

## 5 Conclusion

This study identified three myxobacterial strains—*Cystobacter fuscus* HM-E, *Myxococcus fulvus* KT23, and *Myxococcus fulvus* KE15—with potent broad-spectrum activity against the dominant pathogens of wheat Fusarium Crown Rot (*F. pseudograminearum*, *F. graminearum*, and *F. culmorum*). Through a combination of “wolf-pack” predation, extracellular metabolites, and volatile organic compounds, these strains achieved mycelial inhibition rates of up to 94.84% and significant spore lysis. Greenhouse and field trials validated their superior performance, with field biocontrol efficacies reaching up to 74.67%, significantly outperforming chemical fungicides. Furthermore, the application of these strains promoted plant growth and increased wheat yields by up to 27.82%. These findings establish myxobacteria as a robust, multi-functional microbial resource and provide a sustainable new paradigm for the integrated management of Fusarium Crown Rot in wheat production. Nevertheless, several key aspects remain to be elucidated through further investigation, including the biochemical nature of the antifungal compounds produced by these myxobacteria, the quantifiable contribution of VOCs under field conditions, the molecular mechanisms underlying their growth-promoting effects, and the ecological adaptability of their formulations across diverse pedoclimatic regions.

## Data Availability

The original contributions presented in the study are publicly available. This data can be found at: https://www.ncbi.nlm.nih.gov/nuccore/PX726399.1; https://www.ncbi.nlm.nih.gov/nuccore/PX671533.1; https://www.ncbi.nlm.nih.gov/nuccore/PX671532.1.
