# Supplementary material for: Laboratory screening and field validation of Myxococcus fulvus and Cystobacter fuscus for the biocontrol of wheat Fusarium Crown Rot
Source: Front Microbiol. 2026 Apr 10;17:1804789. doi: 10.3389/fmicb.2026.1804789 (PMC13106548; doi:10.3389/fmicb.2026.1804789)
Supplement: Supplementary file 1 [file Table_1.docx]

**Appendix Table 1 | Field plot experimental design and treatments**

| No. | Treatment | Treatment description |
| --- | --- | --- |
| 1 | HM-E fermentation broth | At the wheat jointing and flowering stages, a 10-fold diluted myxobacterial fermentation broth was sprayed onto the basal stems at 1.5 L per plot. |
| 2 | KT23 fermentation broth | Same application method and timing as HM-E fermentation broth. |
| 3 | KE15 fermentation broth | Same application method and timing as HM-E fermentation broth. |
| 4 | HM-E solid inoculant | At sowing, 420 g per plot of solid inoculant was evenly applied into the seed furrow, lightly covered with soil, and then sown. |
| 5 | KT23 solid inoculant | Same application method as HM-E solid inoculant. |
| 6 | KE15 solid inoculant | Same application method as HM-E solid inoculant. |
| 7 | Sterile fermentation substrate | At sowing, 420 g per plot of sterile solid fermentation substrate was evenly applied into the seed furrow, lightly covered with soil, and then sown. |
| 8 | Tebuconazole | At the wheat jointing and flowering stages, tebuconazole (500-fold dilution) was sprayed onto the basal stems at 1.5 L per plot. |
| 9 | CK (pathogen control) | At the wheat jointing and flowering stages, water was sprayed onto the basal stems at 1.5 L per plot. |
| 10 | Mock | No pathogen inoculation and no application of fungicides, microbial agents, or fertilizers. |
