# Supplementary material for: Laboratory screening and field validation of Myxococcus fulvus and Cystobacter fuscus for the biocontrol of wheat Fusarium Crown Rot
Source: Front Microbiol. 2026 Apr 10;17:1804789. doi: 10.3389/fmicb.2026.1804789 (PMC13106548; doi:10.3389/fmicb.2026.1804789)
Supplement: Supplementary file 2 [file Table_2.docx]

**Appendix Table 2 | Antifungal activity of 81 myxobacterial strains against *Fusarium pseudograminearum* on plates**

| Strain No. | Inhibition rate (%) | Strain No. | Inhibition rate (%) | Strain No. | Inhibition rate (%) |
| --- | --- | --- | --- | --- | --- |
| HM-E | 94.94±1.42 a | NSD58 | 62.82±1.92 ghi | NSD35 | 36.56±1.82 stu |
| KE15 | 92.73±0.75 a | NSE1 | 60.38±1.40 hij | WCH05 | 25.04±5.33 stuv |
| KT23 | 92.57±1.94 a | NSE35 | 58.72±1.77 ijk | NSE32 | 33.39±5.98 tuvw |
| NSE3 | 86.24±4.97 b | WT38 | 57.90±4.70 jk | NSE3 | 32.76±2.06 uvw |
| NST47 | 85.56±1.96 bc | TXE1 | 57.48±1.65 jk | WE9 | 31.43±0.47 vw |
| WST3 | 83.63±1.47 bcd | WCH03 | 57.46±1.94 jk | WE33 | 29.97±1.07 wx |
| WD17 | 81.84±1.65 cd | TXD1 | 55.49±0.79 kl | NSE15 | 29.24±0.56 wx |
| NSD29 | 81.80±0.68 cd | NSD72 | 54.36±2.98 klm | WT7 | 25.98±1.12 xy |
| WY3 | 81.59±1.72 cd | NSD9+27 | 54.26±0.57 klm | KY01 | 25.61±0.47 xy |
| NSD44-3 | 81.31±0.71 cd | NSE46 | 52.77±0.28 lm | WT6 | 25.55±0.18 xy |
| WE17 | 80.44±2.00 d | NST36 | 52.73±1.66 lm | WD7 | 22.24±0.85 yz |
| WE3 | 80.28±2.78 d | NSD30 | 52.11±3.38 lmn | NSD6 | 22.09±1.86 yz |
| KT26 | 80.01±0.97 d | NSD71 | 50.84±0.77 mno | NST55 | 21.70±0.77 yz |
| WE2 | 79.84±0.89 d | NSE30 | 50.70±7.29 mno | MD202 | 20.32±0.63 zA |
| NST49 | 79.08±1.30 d | NSD44 | 48.01±1.01 nop | NSE23 | 20.02±1.96 zA |
| NSE30 | 73.73±2.00 e | WD9 | 47.48±1.56 op | MT404 | 19.49±0.89 zA |
| NST21 | 69.21±5.26 f | NSD202 | 45.73±4.55 pq | NSE49 | 19.31±0.66 zA |
| HN01 | 69.12±1.76 f | NSD34 | 45.67±4.65 pq | WE41 | 18.70±0.47 zA |
| NST34 | 68.95±5.72 f | KE3 | 42.88±5.84 qr | WE47 | 16.31±0.90 A |
| WD12 | 68.46±1.96 f | ME276 | 42.14±2.17 qr | NSD23 | - |
| WE7-1 | 67.84±3.34 f | NST59 | 39.49±2.00 rs | NSE34 | - |
| NST12 | 67.50±5.15 f | NSE72 | 39.30±1.46 rs | WD47 | - |
| NST15 | 67.40±3.67 f | NSE25 | 37.57±1.27 st | WE31 | - |
| NSE2 | 65.70±3.96 fg | NSE36 | 37.36±1.44 stu | WT1 | - |
| NSE16 | 65.14±1.68 fg | NSE44-3 | 37.29±0.34 stu | WE6 | - |
| NSE31 | 65.12±1.50 fg | WD20 | 36.94±0.77 stu | NST35 | - |
| NSE41 | 64.55±2.58 fgh | MT204 | 36.59±1.74 stu | YHD05 | - |

Note: Inhibition rate (%) was determined using the plate confrontation assay against *Fusarium pseudograminearum*. Data were presented as mean±standard deviation, data with the different letters in the same column indicated significantly different (*P*＜0.05).
